# Activation of Glutamatergic Neurons in the Supramammillary Nucleus Promotes the Recovery of Consciousness under Sevoflurane Anesthesia

**DOI:** 10.1002/advs.202406959

**Published:** 2025-04-01

**Authors:** Jiayan Li, Yehui Wu, Yihan Wang, Yumin Wu, Rong Hu, Si Long, Wenqi Huang, Liming Nie, Zhongxing Wang

**Affiliations:** ^1^ Department of Anesthesiology The First Affiliated Hospital Sun Yat‐sen University Guangzhou 510000 China; ^2^ Medical Research Institute Guangdong Provincial People's Hospital (Guangdong Academy of Medical Sciences) Southern Medical University Guangzhou 510000 China

**Keywords:** consciousness, glutamatergic neurons, mechanism of sevoflurane anesthesia, medial septum, supramammillary nucleus

## Abstract

Volatile anesthetics have been widely applied during surgery, but the potential mechanisms by which they influence loss of consciousness (LOC), anesthesia maintenance, and recovery of consciousness (ROC) from anesthesia remain largely unknown. Recent studies have suggested that anesthesia‐induced unconsciousness may be due to specific interactions between neural circuits that regulate sleep and wakefulness. Supramammillary (SuM) glutamatergic neurons are essential for sleep‐wakefulness regulation. However, whether SuM glutamatergic neurons are involved in the modulation of consciousness under sevoflurane anesthesia is unclear. Here, it is shown that the activity of SuM glutamatergic neurons decreased prior to sevoflurane‐induced LOC and gradually increased following ROC. Selective lesioning of SuM glutamatergic neurons promoted the induction of and delayed emergence from sevoflurane anesthesia and increased sevoflurane sensitivity. In addition, optogenetic stimulation of SuM glutamatergic neurons or the SuM‐MS projection promoted behavioral arousal and cortical activation under steady‐state sevoflurane anesthesia (SSSA) and reduced the depth of anesthesia and caused cortical arousal under sevoflurane‐induced burst‐suppression conditions. Collectively, these results provide compelling evidence that SuM glutamatergic neurons contribute to regulating states of consciousness under sevoflurane anesthesia.

## Graphical abstract

Selective lesioning of SuM glutamatergic neurons promoted the induction of and delayed emergence from sevoflurane anesthesia and increased sevoflurane sensitivity. Optogenetic stimulation of SuM glutamatergic neurons or the SuM‐MS projection promoted behavioral arousal and cortical activation under steady‐state sevoflurane anesthesia and reduced the depth of anesthesia under sevoflurane‐induced burst‐suppression conditions.

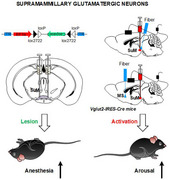

## 1 Introduction

Various kinds of general anesthetics can induce a transient coma‐like state.^[^ ^1^ ^]^ Rapid emergence from general anesthesia is important for modern surgery, but no existing drugs can actively reverse the effects of general anesthesia. Moreover, the exact neural mechanism involved in emergence from general anesthesia‐induced unconsciousness remains unknown.

Accumulating evidence suggests that general anesthetics induce loss of consciousness (LOC) by acting on specific neural substrates that regulate sleep and wakefulness, and activation of some arousal‐promoting brain areas can induce arousal under general anesthesia.^[^ ^2^ ^]^ The supramammillary nucleus (SuM), located in the ventromedial posterior hypothalamus, has recently been regarded as a crucial hub for behaviors that rely on heightened arousal, such as learning, memory, motivation, and locomotion.^[^ ^3^, ^4^ ^]^ Recent studies have also suggested that the SuM is a key node for promoting wakefulness and that the activation of SuM glutamatergic neurons, but not GABAergic neurons, effectively drives wakefulness.^[^ ^5^ ^]^ However, whether SuM glutamatergic neurons participate in regulating states of consciousness under general anesthesia remains unclear. Additionally, the SuM not only extensively innervates multiple brain areas that have been reported to promote emergence from general anesthesia, such as the paraventricular thalamus,^[^ ^3^, ^6^, ^7^ ^]^ locus coeruleus,^[^ ^5^, ^8^, ^9^ ^]^ ventral tegmental area,^[^ ^3^, ^10^, ^11^ ^]^ and medial prefrontal cortex,^[^ ^3^, ^12^, ^13^ ^]^ but also has substantial reciprocal connections with arousal‐promoting areas related to general anesthesia, including the medial septum,^[^ ^3^, ^14^, ^15^ ^]^ basal forebrain,^[^ ^5^, ^16^, ^17^ ^]^ lateral hypothalamus^[^ ^3^, ^18^, ^19^ ^]^ and dorsal raphe.^[^ ^3^, ^20^, ^21^ ^]^ Therefore, we hypothesized that SuM glutamatergic neurons play a critical role in regulating states of consciousness during general anesthesia.

The medial septum (MS), a key hub for the regulation of arousal‐based behaviors and the maintenance of wakefulness, has recently been reported to modulate states of consciousness during sevoflurane anesthesia.^[^ ^14^ ^]^ Previous studies have also demonstrated that SuM glutamatergic neurons can monosynaptically innervate MS glutamatergic neurons by releasing glutamate^[^ ^22^, ^23^ ^]^ and that stimulation of the axonal terminals of the SuM‐MS pathway induces a rapid transition from sleep to wakefulness.^[^ ^24^ ^]^ Thus, we hypothesized that activation of glutamatergic SuM neurons, by projecting to the MS, plays a role in modulating the states of consciousness in propofol anesthesia.

In our study, we found that population activities of glutamatergic SuM neurons decreased after sevoflurane induction and gradually increased during the sevoflurane emergence period, as determined via a combination of in vivo fiber photometry and electroencephalogram(EEG)/ electromyography (EMG) recordings. Selective lesioning of SuM glutamatergic neurons accelerated the induction time and prolonged the emergence time from sevoflurane anesthesia. Finally, optogenetic stimulation of glutamatergic SuM neurons or the SuM‐MS projection robustly induced behavioral emergence and cortical activation during steady‐state sevoflurane anesthesia (SSSA) and reduced the depth of anesthesia during deep anesthesia. These results provide compelling evidence that SuM glutamatergic neurons contribute to regulating states of consciousness under sevoflurane anesthesia.

## 2 Results

### 2.1 Glutamatergic Neurons in the SuM Respond to Sevoflurane Anesthesia

**Figure 1:**
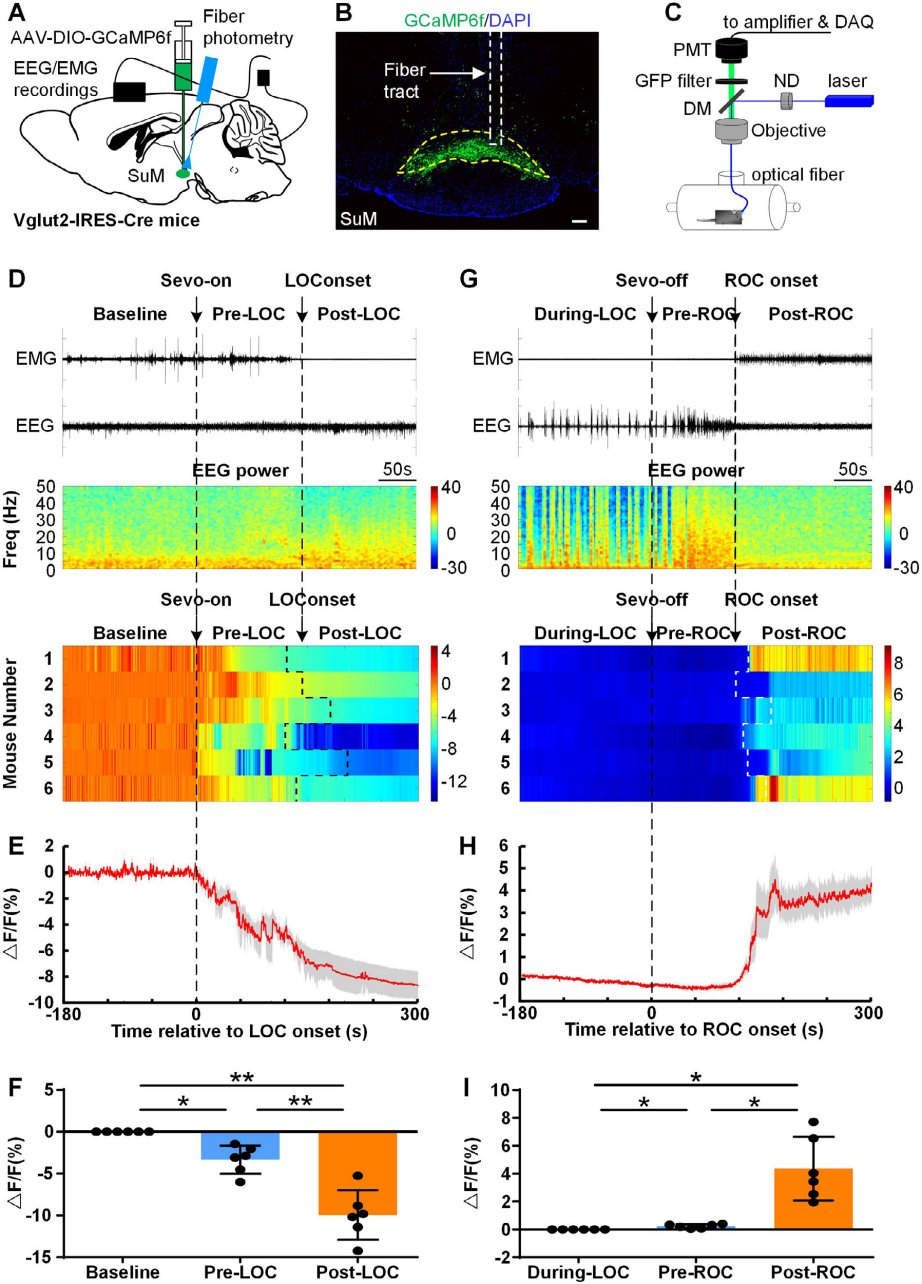
Changes in the activity of SuM glutamatergic neurons in response to the induction of and emergence from sevoflurane anesthesia in mice. A) Schematic of rAAV‐EF1α‐DIO‐GCaMP6f injected into the SuM of Vglut2‐Cre mice. B) Representative image of GCaMP6f (green) localized to the SuM of Vglut2‐Cre mice and track of the optic fiber implanted above the SuM; scale bar, 200 µm. C) Schematic of the EEG/EMG recording process and fiber photometry setup. D) Top: Example EEG/EMG traces and EEG power spectrograms during sevoflurane induction. Bottom: Heatmap of Ca^2+^ signals aligned to LOC transitions (*n* = 6 mice). E) The mean Ca^2+^ transients ± SEM (shaded area) for SuM glutamatergic neurons during sevoflurane‐induced LOC (*n* = 6 mice). F) Quantification of Ca^2+^ signal changes in three phases: baseline, pre‐LOC, and post‐LOC. The data are shown as the mean ± SD, ^*^ *P *< 0.05, ^**^ *P* < 0.01, n = 6 mice, one‐way repeated measures ANOVA with post hoc Tukey's test. G) Top: Representative EEG/EMG traces and EEG power spectrograms during sevoflurane emergence. Bottom: Heatmap of Ca^2+^ signals aligned to ROC transitions (n = 6 mice). H) The mean Ca^2+^ transients ± SEM (shaded area) for SuM glutamatergic neurons during ROC transitions (n = 6 mice). I) Quantification of Ca^2+^ signal changes in three consecutive periods: during LOC, pre‐ROC, and post‐ROC. The data are shown as the mean ± SD, ^*^ *P* < 0.05, n = 6 mice, one‐way repeated measures ANOVA with post hoc Tukey's test.

To determine whether the activity of SuM glutamatergic neurons is correlated with sevoflurane administration, AAV‐Ef1α‐DIO‐GCaMP6f was microinjected into the SuM of Vglut2‐Cre mice, and fiber photometry was used to detect changes in the Ca^2+^ signals of SuM glutamatergic neurons (**Figure** **1**A–C). We explored the real‐time activities of SuM glutamatergic neurons during induction and emergence from 2.4% sevoflurane anesthesia. For the sevoflurane induction period, the Ca^2+^ signals were divided into three phases: the pre‐anesthesia baseline (baseline, from −180 to 0 s, 0 s was the time point at which sevoflurane was administered), the initial period before LOC (pre‐LOC, from 0 s to LOC) and the early period after LOC (post‐LOC, from LOC to 300 s). As shown in Figure 1D–F, the Ca^2+^ signals decreased during the early period before LOC (pre‐LOC vs baseline, −3.3 ± 1.7%, *P* = 0.010; Figure 1F). The Ca^2+^ signals decreased further after LOC compared with those at baseline (post‐LOC vs baseline, −10.0 ± 3.0%, *P* = 0.001; Figure 1F). For the sevoflurane emergence period, we also divided the Ca^2+^ signals into three phases: during‐LOC(baseline), from −180 to 0 s (the unconsciousness anesthesia state, 0 s was the time point when sevoflurane was turned off); pre‐recovery of consciousness (pre‐ROC, from 0 s to ROC); and the early period after ROC (post‐ROC, from ROC to 300 s). As shown in Figure 1G–I, compared with those in the unconsciousness anesthesia state, the Ca^2+^ signals slightly increased in the early period before ROC (pre‐ROC vs during‐LOC, 0.2 ± 0.1%, *P* = 0.009; Figure 1I) and then robustly increased after ROC (after‐ROC vs during‐LOC, 4.4 ± 2.3%, *P* = 0.006; Figure 1I).

Overall, we found that the Ca^2+^ signals of SuM glutamatergic neurons were suppressed during the sevoflurane induction period but recovered during the early emergence period, suggesting that SuM glutamatergic neurons participate in anesthetic state transitions under sevoflurane anesthesia.

### 2.2 Genetic Ablation of Glutamatergic Neurons in the SuM Promotes the Induction of and Prolongs Recovery from Sevoflurane General Anesthesia

**Figure 2:**
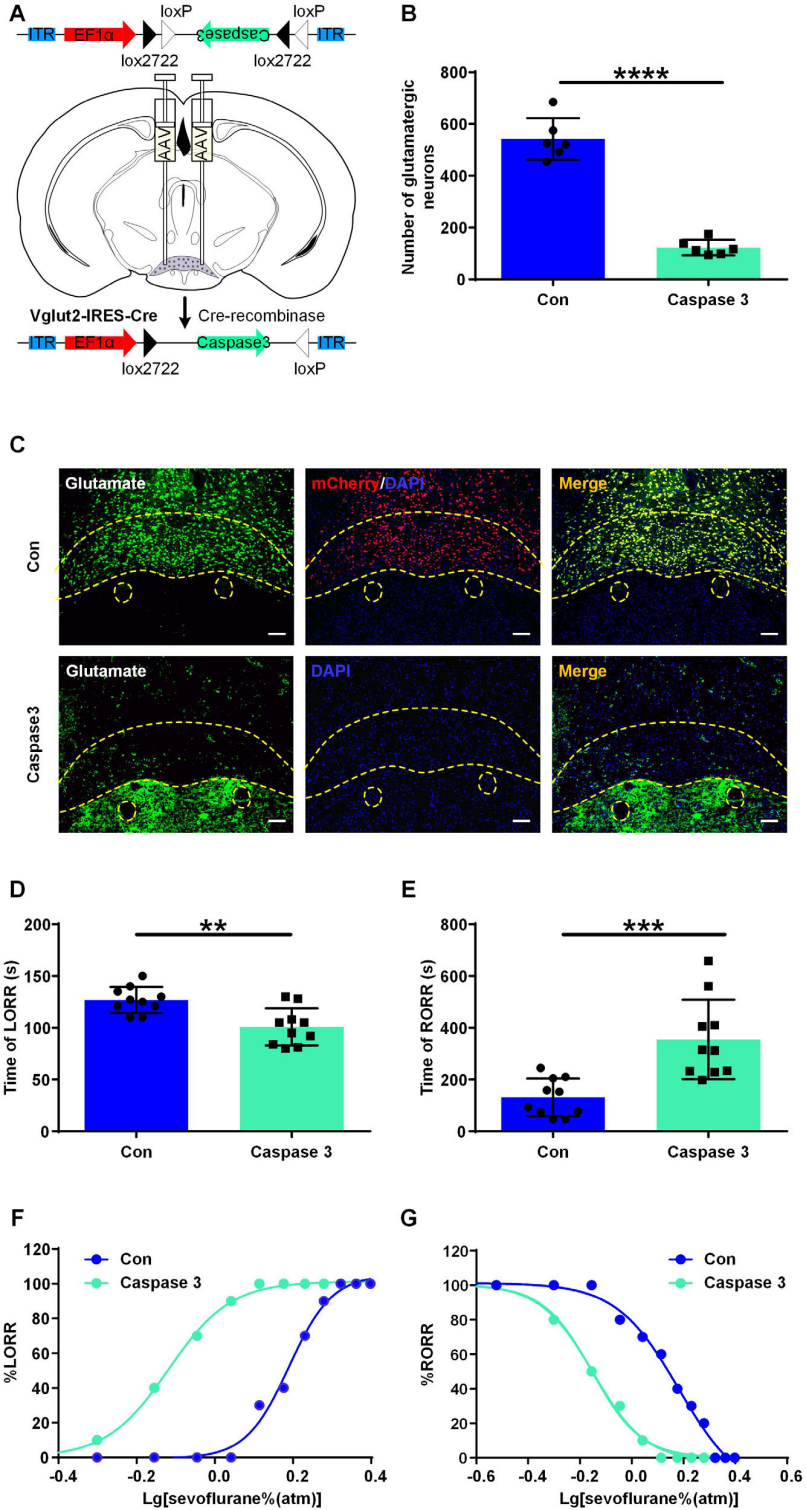
Selective lesioning of SuM glutamatergic neurons accelerates sevoflurane induction, delays sevoflurane emergence, and increases sensitivity to anesthesia. A) Schematic of rAAV‐EF1α‐DIO‐taCasp3‐T2A‐TEVp injection into the SuM of Vglut2‐Cre mice. B) Number of SuM glutamatergic neurons in the control and Caspase 3 groups. The data are shown as the mean ± SD, ^****^ *P* < 0.0001, unpaired Student's *t*‐test, n = 6 mice. C) Representative image of glutamate (green)/DAPI (blue) immunofluorescence for mice in the control and Caspase 3 groups. Scale bar, 100 µm. D) The induction time after exposure to 2.4% sevoflurane (1 MAC) in the control and Caspase 3 groups. The data are shown as the mean ± SD, ^**^ *P* < 0.01, unpaired Student's *t*‐test. n = 10 mice. E) The emergence time after exposure to 2.4% sevoflurane (1 MAC) for 30 min in the control and the caspase 3 groups. The data are shown as the mean ± SD, ^***^ *P* < 0.001, unpaired Student's *t*‐tests, n = 10 mice. F) The dose‐response curve of the percent LORR (n = 10 mice). G) The dose‐response curve of the percent RORR (n = 10 mice).

Next, to explore the role of SuM glutamatergic neurons during sevoflurane anesthesia, a Cre‐dependent AAV expressing Caspase 3 (rAAV‐EF1α‐DIO‐taCasp3‐T2A‐TEVp) was bilaterally microinjected into the SuM of Vglut2‐Cre mice to selectively lesion glutamatergic neurons, and AAV‐EF1α‐DIO‐mCherry was microinjected into the SuM of the mice in the control group (**Figure** **2**A). The immunochemical results confirmed that the number of SuM glutamatergic neurons decreased from 542.0 ± 80.6 to 122.5 ± 30.3 after virus injection (*P* < 0.0001, n = 6 mice, Figure 2B,C). Compared with the control group, in the SuM glutamatergic neuron lesion group, the induction time significantly decreased from 126.9 ± 12.6 s to 100.8 ± 17.9 s (*P* = 0.001, n = 10 per group, Figure 2D), and the emergence time markedly increased from 131.0 ± 72.9 s to 355.3 ± 153.9 s (*P* = 0.001, n = 6 per group, Figure 2E). Furthermore, as shown in Figure 2F,G, compared with those in the control group, the dose‐response curves for both the loss of righting reflex (LORR) and recovery of righting reflex (RORR) in the Caspase 3 group were shifted to the left. The minimum alveolar concentration (MAC) for the LORR (MAC_LORR_) was significantly lower in the Caspase 3 group than in the control group (mean [95% CI], 0.76 [0.74 to 0.78] vs 1.55 [1.48 to 1.6] vol%, *P* < 0.0001, Figure 2F and Table S1, Supporting Information) and the MAC for the RORR (MAC_RORR_) also decreased (Caspase 3 vs control, 0.71 [0.67 to 0.74] vs 1.52 [1.31 to 1.75] vol%, *P* = 0.001, Figure 2G and Table S1, Supporting Information). These results suggest that selective ablation of SuM glutamatergic neurons facilitates the induction of sevoflurane anesthesia, prolongs emergence from sevoflurane anesthesia, and increases sensitivity to sevoflurane in mice.

### 2.3 Selective Lesioning of SuM Glutamatergic Neurons Suppresses Cortical Arousal from Sevoflurane Anesthesia

**Figure 3:**
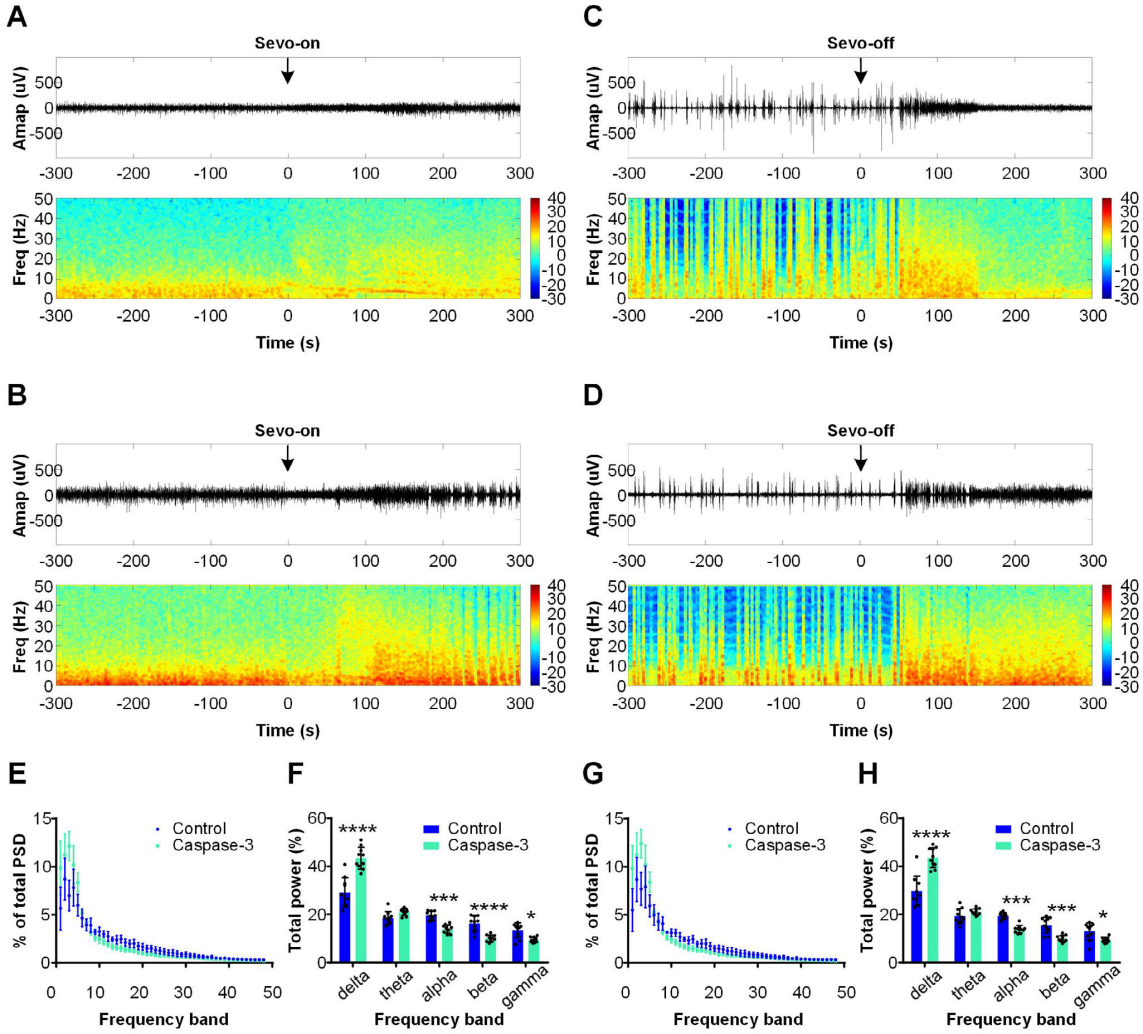
Selective ablation of SuM glutamatergic neurons promotes sevoflurane‐induced cortical sedation and suppresses cortical arousal during sevoflurane emergence. A, B) Representative images of raw EEG traces (top) and EEG power spectrograms (bottom) during sevoflurane induction in the control (A) and Caspase 3 groups (B). '0′ indicates the initiation of sevoflurane anesthesia. C, D) Typical examples of raw EEG traces (top) and EEG power spectrograms (bottom) during sevoflurane emergence in the control (C) and Caspase 3 groups (D). '0′ refers to the cessation of sevoflurane anesthesia. E) Normalized power spectral densities (PSDs) of EEG signals during sevoflurane induction in the control (blue) and Caspase 3 (green) groups. EEG signals within 5 min after the start of sevoflurane anesthesia were analyzed. The data are shown as the mean ± SD (n = 10 mice). F) Relative EEG power during sevoflurane induction in the control (blue) and Caspase 3 (green) groups. The data are shown as the mean ± SD, ^*^ *P* < 0.05, ^***^ *P* < 0.001, ^****^ *P* < 0.0001, two‐way ANOVA with post hoc Bonferroni's test, n = 10 mice. G) Normalized PSD of EEG signals during sevoflurane emergence in the control (blue) and Caspase 3 (green) groups. EEG signals within 5 min after the cessation of sevoflurane anesthesia were analyzed. The data are shown as the mean ± SD (n = 10 mice). H) Relative EEG power during sevoflurane emergence in the control (blue) and Caspase 3 (green) groups. The data are shown as the mean ± SD, ^*^ *P* < 0.05, ^***^ *P* < 0.001, ^****^ *P* < 0.0001, two‐way ANOVA with post hoc Bonferroni's test, n = 10 mice).

To further clarify how lesioning SuM glutamatergic neurons affect induction and emergence from sevoflurane anesthesia, we recorded electroencephalogram (EEG) signals in the somatosensory cortex. During the anesthesia induction period, compared with the mice in the control group, the mice in the Caspase 3 group quickly transitioned from an awake‐EEG state to an anesthesia‐EEG state (**Figure** **3**A,B). In addition, power spectral density (PSD) analysis of the EEG data showed that ablation of SuM glutamatergic neurons in Caspase 3 mice significantly increased delta power (Caspase 3 vs control, 43.4% ± 4.6% vs 29.2% ± 6.1%, *P* < 0.0001) and significantly decreased alpha power (13.9% ± 1.7% vs 19.5% ± 1.8%, *P* = 0.0009), beta power (10.0% ± 1.4% vs 16.2% ± 3.2%, *P* = 0.0003) and gamma power (9.4% ± 1.1% vs 13.5% ± 3.0%, *P* = 0.023) compared with those of control mice (Figure 3E,F). During the sevoflurane anesthesia emergence period, compared with those in the control group, the mice in the Caspase 3 group exhibited a delay in the transition from an anesthesia‐EEG state to an awake‐EEG state (Figure 3C,D). Lesioning SuM glutamatergic neurons also substantially increased delta power (caspase 3 vs control, 43.6% ± 4.1% vs 29.8% ± 6.2%, *P* < 0.0001) and decreased alpha power (13.8% ± 1.6% vs 19.3% ± 1.4%, *P* = 0.0005), beta power (9.9% ± 1.3% vs 15.6% ± 3.3%, *P* = 0.0003) and gamma power (9.3% ± 1.1% vs 13.0% ± 3.6%, *P* = 0.03) compared with those of control mice (Figure 3G,H). Overall, these findings suggest that the knockdown of SuM glutamatergic neurons accelerated sevoflurane‐induced sedation and inhibited cortical arousal.

### 2.4 Optogenetic Activation of SuM Glutamatergic Neurons Drives Behavioral Emergence and Cortical Activation during Steady‐State Sevoflurane Anesthesia

**Figure 4:**
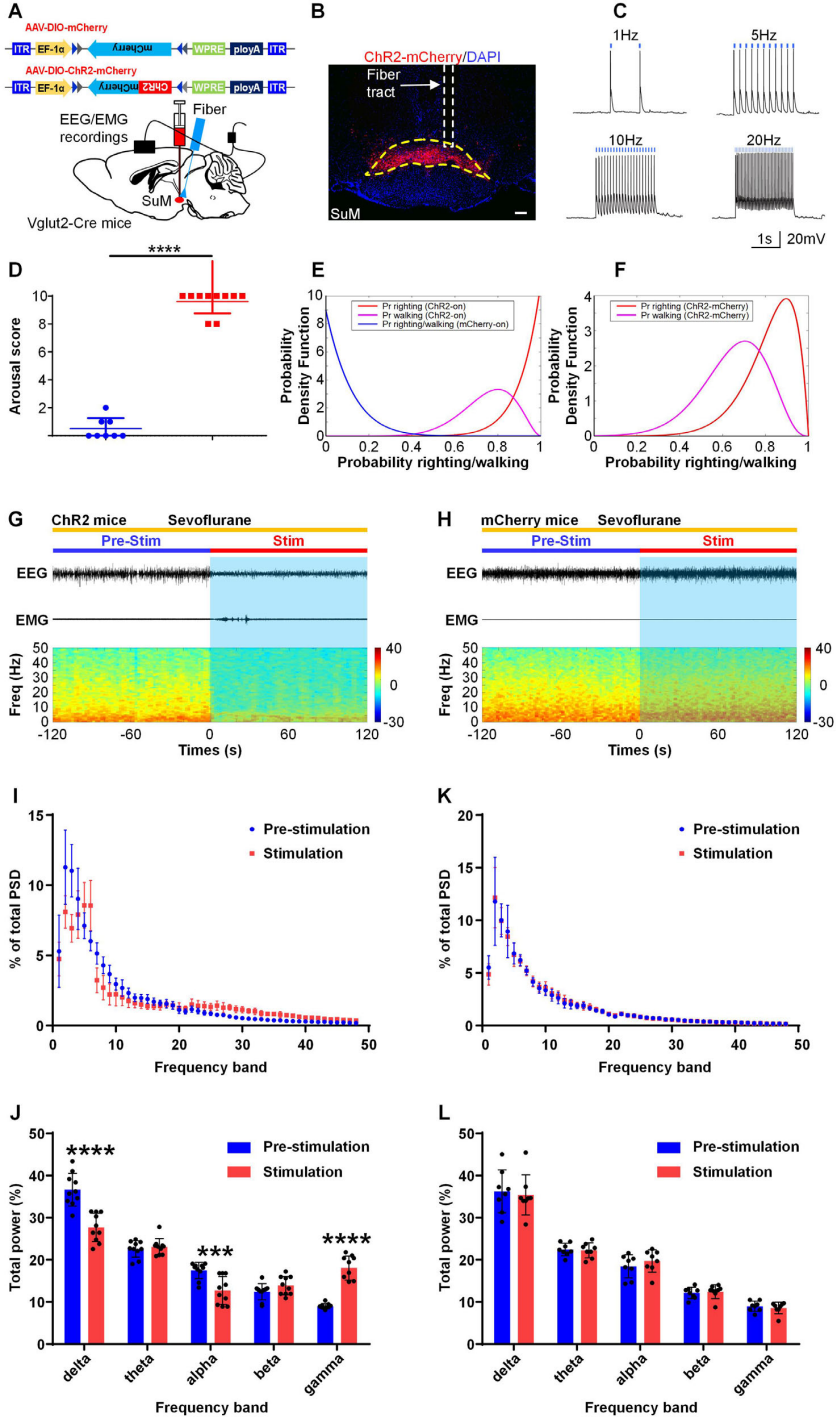
Optogenetic stimulation of SuM glutamatergic neurons elicits arousal behavior and drives cortical activation under steady‐state sevoflurane anesthesia in male mice. A) Schematic showing the injection of AAV‐DIO‐ChR2‐mCherry or AAV‐DIO‐mCherry into the SuM of Vglut2‐Cre mice. B) Typical image showing the expression of ChR2‐mCherry (red) in SuM glutamatergic neurons and the position of the optical fiber (white dotted box) in the SuM. Scale bars, 200 µm. C) Representative traces of neuronal firing in ChR2‐expressing glutamatergic neurons in the SuM evoked by 473‐nm light stimulation at different frequencies. D) Arousal scores for the optical activation of SuM glutamatergic neurons (20 Hz, 5 ms pulses, 120 s) under SSSA. The data are presented as the median ± interquartile range; ^****^ *P* < 0.0001, Mann‐Whitney U‐test, n = 8 in the mCherry‐on group and n = 10 in the ChR2‐on group. E) Posterior distributions for the likelihood of the righting reflex and walking after optical activation of SuM glutamatergic neurons under SSSA. Posterior distributions were derived from the beta distributions. F) The difference in the posterior probability of the righting reflex and walking between mCherry‐on mice and ChR2‐on mice during SSSA (20 Hz, 5 ms pulses, 120 s). G‐H) Typical raw EEG/EMG signals (top) and EEG power spectra (bottom) of ChR2 mice (G) and control mice (H) following acute optical stimulation under SSSA. I) Normalized PSD 120 s before (blue) and 120 s during stimulation of ChR2 mice during SSSA. J) Relative EEG power before (blue) and during (red) optical stimulation of SuM glutamatergic neurons. The data are represented as the mean ± SD; ^***^ *P* < 0.001, ^****^ *P* < 0.0001, two‐way repeated‐measures ANOVA followed by Sidak's post hoc test, n = 10 in each group. K) Normalized PSD 120 s before (blue) and 120 s during stimulation of control mice during SSSA. L) Relative EEG power before (blue) and during (red) stimulation of control mice during SSSA. The data are represented as the mean ± SD, two‐way repeated‐measures ANOVA followed by Sidak's post hoc test, n = 8 in each group.

To investigate whether stimulation of SuM glutamatergic neurons can restore consciousness during maintenance of light anesthesia, AAV‐EF1a‐DIO‐ChR2‐mCherry or AAV‐EF1α‐DIO‐mCherry (as a control) was bilaterally injected into the SuM of Vglut2‐Cre male mice, and an optical fiber was placed above the SuM to selectively activate SuM glutamatergic neurons (**Figure** **4**A).Immunofluorescence staining confirmed that SuM glutamatergic neurons were successfully transfected with the virus expressing ChR2 (Figure 4B). We confirmed the functional expression of ChR2 using the whole‐cell patch‐clamp recordings (Figure 4C). When the male mice lost their righting reflex for 20 min during SSSA, optogenetic stimulation (473 nm, 20 Hz, 5 ms, 120 s) of SuM glutamatergic neurons in the ChR2 mice elicited obvious arousal behavior (Figure 4D and Table S2, Video S1, Supporting Information), including head, leg and tail movements (10/10), the righting reflex (10/10), and walking (8/10), while this arousal behavior was rarely observed in the mCherry‐on group (Figure 4D and Table S2, Video S2, Supporting Information). The Bayesian 95% confidence interval (CI) for the difference in the probability of righting (walking) between the ChR2‐on mice and the control mice under 20 Hz stimulation was 0.528–0.976 (0.314–0.892; Figure 4E). The posterior probability of the difference in righting (walking) between the two groups being greater than 0 under 20 Hz stimulation was 1.0000 (0.9997), which was statistically significant (Figure 4F). In addition, optical stimulation of SuM glutamatergic neurons at 20 Hz induced a quick transition from slow‐wave activity to low‐voltage fast activity in the ChR2‐on group (Figure 4G and Video S1, Supporting Information) but not in the mCherry‐on group (Figure 4H and Video S2, Supporting Information). Power spectral density analysis of the EEG data showed that optical stimulation of SuM glutamatergic neurons significantly decreased delta power (prestimulation vs stimulation, 36.6 ± 3.9% vs 27.7 ± 3.4%, *P* < 0.0001; Figure 4I,J) and alpha power (17.5 ± 1.9% vs 12.7 ± 3.3%, *P* = 0.0008; Figure 4I,J) but markedly increased gamma power (9.0 ± 0.6% vs 18.0 ± 2.8%, *P* < 0.0001; Figure 4I,J). However, this phenomenon was not observed in the control group (Figure 4K,L). Considering sex as a biological variable is now widely recognized as essential in research, and we also performed these experiments in female mice. An AAV‐EF1a‐DIO‐ChR2‐mCherry or AAV‐EF1α‐DIO‐mCherry (as a control) was bilaterally microinjected into the SuM of Vglut2‐Cre female mice, and we also placed an optical fiber above the SuM to selectively activate SuM glutamatergic neurons (Figure S1A, Supporting Information). we confirmed the expression of the virus expressing ChR2 in the SuM glutamatergic neurons (Figure S1B, Supporting Information), and the functional expression of ChR2 was determined by the whole‐cell patch‐clamp recordings in female mice (Figure S1C, Supporting Information). When the female mice lost their righting reflex for 20 min during SSSA, optogenetic stimulation (473 nm, 20 Hz, 5 ms, 120 s) of SuM glutamatergic neurons in the ChR2 mice induced significant arousal behavior (Figure S1D, Table S3, Video S3, Supporting Information), including head, leg and tail movements (8/8), the righting reflex (8/8), and walking (7/8), while this arousal behavior was rarely seen in the control (mCherry‐on) group (Figure S1D, Table S3, Video S4, Supporting Information). The Bayesian 95% confidence interval (CI) for the difference in the probability of righting (walking) between the ChR2‐on group and the control group of female mice under 20 Hz stimulation was 0.465–0.972 (0.331–0.927; Figure S1E, Supporting Information). The posterior probability of the difference in righting (walking) between the two groups being greater than 0 under 20 Hz stimulation was 1.0000 (0.9996), which was statistically significant (Figure S1F, Supporting Information). In addition, optical stimulation of SuM glutamatergic neurons at 20 Hz induced a quick transition from slow‐wave activity to low‐voltage fast activity in the ChR2‐on group (Figure S1G, Video S3, Supporting Information) but not in the mCherry‐on group (Figure S1H, Video S4, Supporting Information). Power spectral density analysis of the EEG data showed that optical stimulation of SuM glutamatergic neurons significantly decreased delta power (prestimulation vs stimulation, 44.5 ± 4.0% vs 31.5 ± 4.8%, *P* < 0.0001; Figure S1I,J, Supporting Information) but markedly increased beta power (9.5 ± 1.6% vs 16.9± 4.1%, *P* = 0.0001; Figure S1I,J, Supporting Information) and gamma power (7.6.0 ± 1.3% vs 14.8 ± 2.7%, *P* = 0.0002; Figure S1I,J, Supporting Information) in female mice. However, this phenomenon was not observed in the control group of female mice (Figure S1K,L, Supporting Information). Together, these findings suggest that the activation of SuM glutamatergic neurons was sufficient to induce behavioral arousal and cortical activation under SSSA.

### 2.5 Optogenetic Activation of SuM Glutamatergic Neurons Reduces the Depth of Anesthesia during Sevoflurane‐Induced Burst Suppression

**Figure 5:**
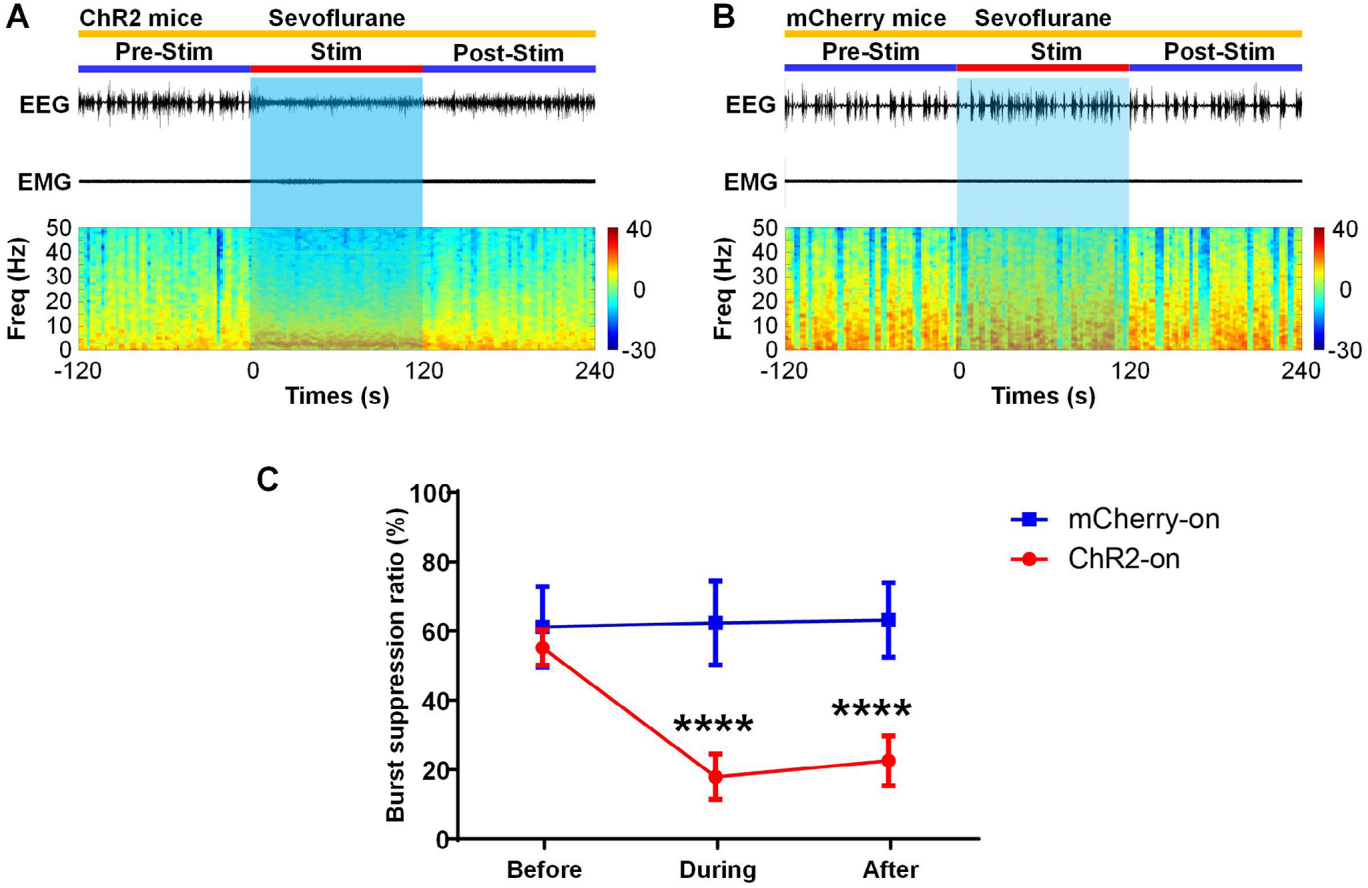
Activation of SuM glutamatergic neurons induces cortical activation and reduces the depth of anesthesia under deep anesthesia. A, B) An example showing raw EEG/EMG traces (top) and EEG power spectra (bottom) in ChR2 mice (A) and control mice (B) following acute optical stimulation under sevoflurane‐induced burst suppression conditions. C) The BSR was analyzed 120 s before (blue), 120 s during (red), and 120 s after (blue) optical stimulation under EEG burst suppression conditions at 20 Hz in ChR2 mice and control mice. The data are shown as the mean ± SD, ^****^ *P* < 0.0001, two‐way ANOVA followed by Sidak's post hoc test, n = 10 in the ChR2 group and n = 8 in the control group.

We further explored the effects of SuM glutamatergic neuron activation on states of consciousness during burst suppression oscillations, which are observed under the maintenance of deep anesthesia. We found that optical stimulation of SuM glutamatergic neurons at 20 Hz caused a rapid decrease in the duration of the suppression period in the burst‐suppression state induced by 2.5% sevoflurane in ChR2‐on mice (**Figure** **5**A and Video S5, Supporting Information). However, there were no significant changes in the EEG of the mCherry‐on mice (Figure 5B and Video S6, Supporting Information). Stimulation of SuM glutamatergic neurons significantly reduced the burst‐suppression ratio (prestimulation vs stimulation, 56.2 ± 5.1% vs 18.9 ± 6.6%, *P* < 0.0001; Figure 5C). After 120 s of stimulation, the BSR gradually increased but was still lower than that before stimulation (poststimulation vs prestimulation, 23.5 ± 7.2% vs 56.2 ± 5.1%; *P* < 0.0001, Figure 5C). However, there were no significant differences in the burst‐suppression ratio (Figure 5C) in the mCherry‐on mice during or after optical stimulation compared with those of the prestimulation period. Thus, these data indicate that the activation of SuM glutamatergic neurons can induce cortical activation and reduce the depth of anesthesia during deep anesthesia.

### 2.6 SuM Glutamatergic Neurons Promote Behavioral and Cortical Arousal under Sevoflurane Anesthesia through the SuM‐MS Pathway

**Figure 6:**
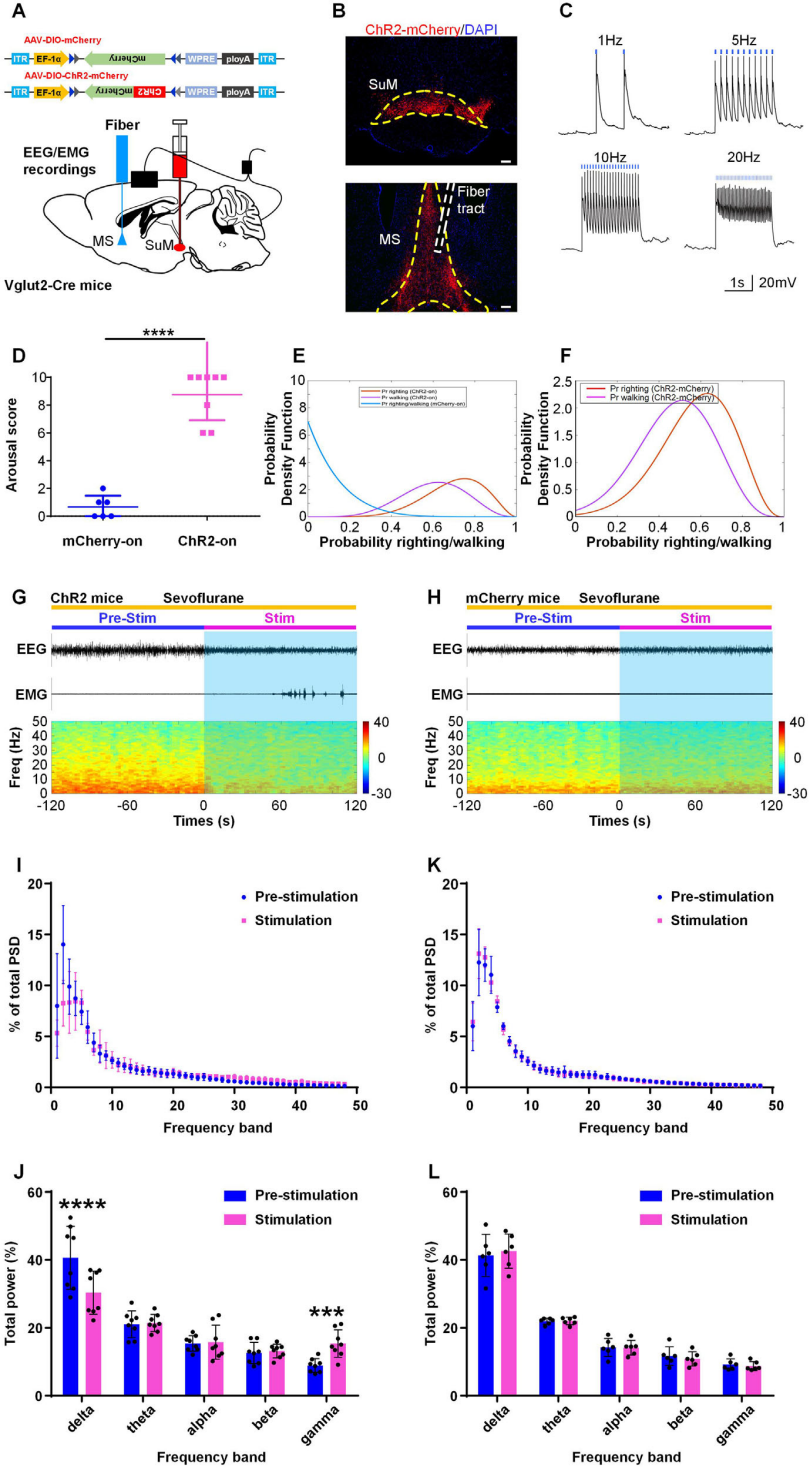
Optogenetic stimulation of the SuM‐MS projection induces arousal behavior and drives cortical activation under steady‐state sevoflurane anesthesia. A) Schematic showing the injection of AAV‐DIO‐ChR2‐mCherry or AAV‐DIO‐mCherry into the SuM of Vglut2‐Cre mice, and the optical fiber was placed above the MS. B) Typical image showing the expression of ChR2‐mCherry (red) in SuM glutamatergic neurons (Top) and the position of the optical fiber (white dotted box) in the MS (Bottom). Scale bars, 200 µm. C) Blue light pulses (5 ms pulses) elicited action potentials in ChR2‐expressing SuM glutamatergic neurons at different frequencies. D) Arousal scores for the optical activation of the SuM‐MS pathway (20 Hz, 5 ms pulses, 120 s) under SSSA. The data are presented as the median ± interquartile range; ^****^ *P* < 0.0001, Mann‐Whitney U‐test, *n* = 6 in the mCherry‐on group and n = 8 in the ChR2‐on group. E) Posterior distributions for the likelihood of the righting reflex and walking after optical activation of the SuM‐MS pathway under SSSA. Posterior distributions were derived from the beta distributions. F) The difference in the posterior probability of the righting reflex and walking between mCherry‐on mice and ChR2‐on mice during SSSA (20 Hz, 5 ms pulses, 120 s). G, H) Typical raw EEG/EMG signals (top) and EEG power spectra (bottom) of ChR2 mice (G) and control mice (H) following acute optical stimulation under SSSA. I) Normalized PSD 120 s before (blue) and 120 s during stimulation of the SuM‐MS pathway in ChR2 mice during SSSA. J) Relative EEG power before (blue) and during (red) optical stimulation of the SuM‐MS pathway. The data are represented as the mean ± SD; ^***^ *P* < 0.001, ^****^ *P* < 0.0001, two‐way repeated‐measures ANOVA followed by Sidak's post hoc test, n = 8 in each group. K) Normalized PSD 120 s before (blue) and 120 s during stimulation of control mice during SSSA. L) Relative EEG power before (blue) and during (red) stimulation of control mice during SSSA. The data are represented as the mean ± SD, two‐way repeated‐measures ANOVA followed by Sidak's post hoc test, n = 6 in each group.

**Figure 7:**
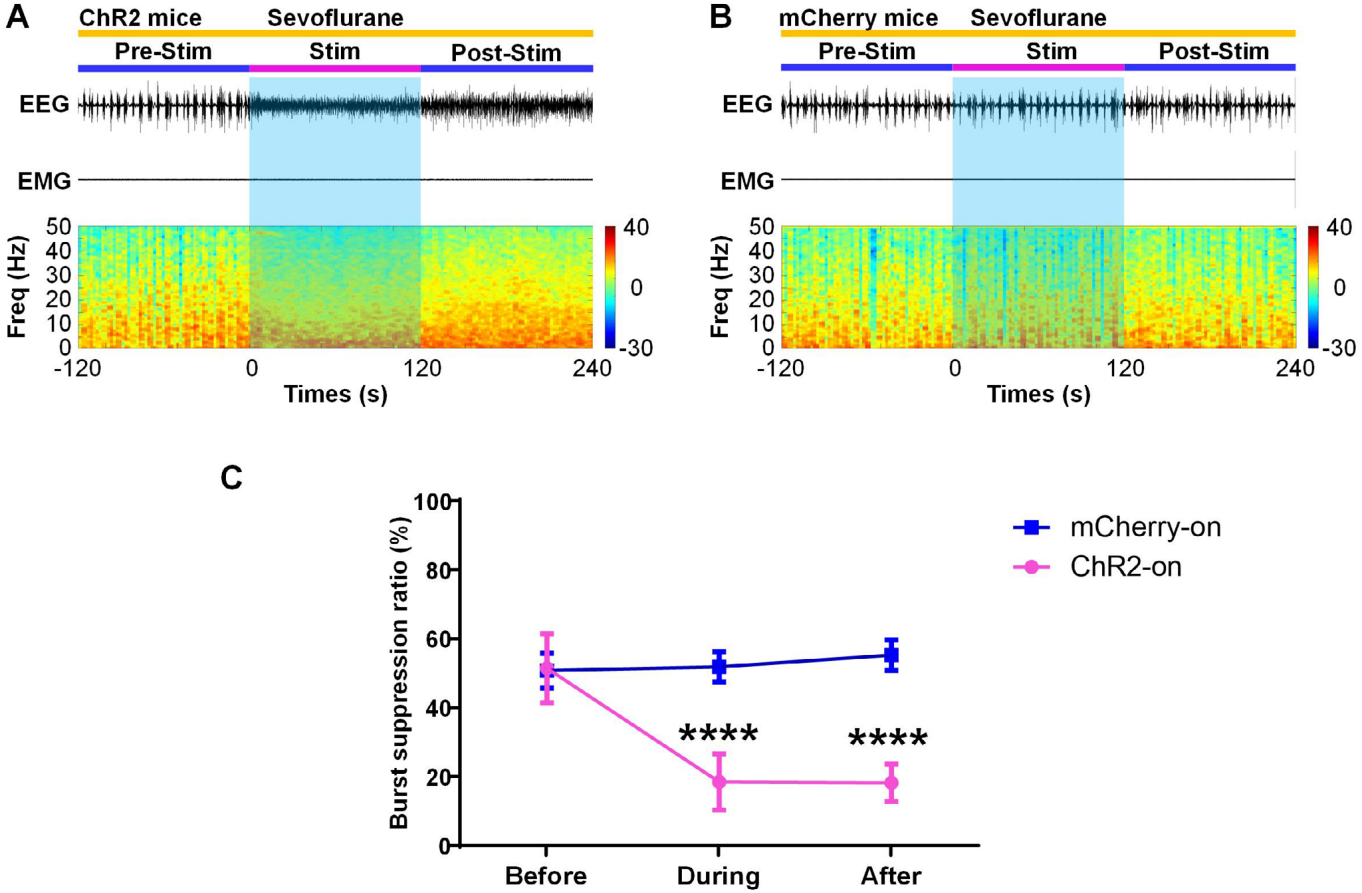
Activation of the SuM‐MS projection drives cortical activation and reduces the depth of anesthesia under deep anesthesia. A, B) An example showing raw EEG/EMG traces (top) and EEG power spectra (bottom) in ChR2 mice (A) and control mice (B) following acute optical stimulation of the SuM‐MS projection under sevoflurane‐induced burst suppression state. C) The BSR was analyzed 120 s before (blue), 120 s during (red), and 120 s after (blue) optical stimulation of the SuM‐MS projection under EEG burst suppression conditions at 20 Hz in ChR2 mice and control mice. The data are shown as the mean ± SD; ^****^ *P* < 0.0001, two‐way ANOVA followed by Sidak's post hoc test, n = 6 in the mCherry‐on group and n = 8 in the ChR2‐on group.

To determine whether the SuM‐MS pathway plays a critical role in the modulation of sevoflurane anesthesia‐induced unconsciousness, we injected the AAV‐hSyn‐DIO‐mCherry or AAV‐hSyn‐DIO‐mCherry virus into the SuM of vglut2‐Cre mice and implanted the optical fiber above the MS (**Figure** **6**A). As shown in Figure 6B, robust mCherry expression was observed in cell bodies within SuM, and the axonal terminals in MS were also labeled with mCherry, suggesting SuM glutamatergic neurons send dense projections to the MS. The functional expression of ChR2 was determined by the whole‐cell patch‐clamp recordings (Figure 6C). Activation of the SuM‐MS pathway at 20 Hz in ChR2 mice induced behavioral arousal (Figure 6D and Table S4, Video S7, Supporting Information), including head, leg, and tail movements (8/8), righting (6/8), and walking (5/8), during steady‐state anesthesia with sevoflurane, which was scarcely observed in mCherry‐on mice (Figure 6D and Table S4, Video S8, Supporting Information). The Bayesian 95% CI for the difference in the probability of righting (walking) between the ChR2‐on mice and the control mice under 20 Hz stimulation was 0.185–0.865(0.081–0.795; Figure 6E). The posterior probability of the difference in righting (walking) between the two groups being greater than 0 under 20 Hz stimulation was 0.9969 (0.9895), which was statistically significant (Figure 6F). We also found that optical stimulation of SuM^vglut2+^ terminals in the MS at 20 Hz in ChR2 mice induced a rapid transition from slow‐wave activity to low‐voltage fast activity (Figure 6G), but in mCherry‐on mice (Figure 6H). Power spectral density analysis of the EEG data showed that optical activation of the SuM‐MS projection significantly decreased delta power (prestimulation vs stimulation, 40.6 ± 9.3% vs 30.4 ± 6.4%, *P* < 0.0001; Figure 6I,J) and increased the gamma power (8.9 ± 2.0% vs 15.4 ± 4.0%, *P* = 0.0001; Figure 6I,J), whereas these changes were not seen in the control group (Figure 6K,L). In addition, during the sevoflurane‐induced burst suppression state, activation of the SuM‐MS pathway caused a rapid decrease in the EEG suppression period (**Figure** **7**A and Video S9, Supporting Information) and decreased BSR (prestimulation vs stimulation, 52.4 ± 10.0% vs 19.4 ± 8.2%, *P* < 0.0001; Figure 7C). However, there were no significant differences in the EEG (Figure 7B and Video S10, Supporting Information) or BSR (Figure 7C) during optical stimulation compared with those of the prestimulation period in the control mice. Taken together, these results suggest that activation of the SuM‐MS pathway is capable of inducing behavioral and cortical arousal during steady‐state sevoflurane anesthesia and reducing the depth of anesthesia during deep anesthesia.

## 3 Discussion

In this study, through in vivo fiber photometry, EEG‐EMG, behavioral tests, selective ablation, and optogenetic activation experiments, we found that SuM glutamatergic neurons promote arousal under sevoflurane anesthesia. Moreover, we found that the Ca^2+^ signals of SuM glutamatergic neurons decreased after sevoflurane induction and gradually increased during the sevoflurane emergence period. Selective lesioning of SuM glutamatergic neurons reduced the induction time and prolonged the emergence time from sevoflurane anesthesia. In addition, optogenetic activation of SuM glutamatergic neurons or the SuM‐MS projection effectively induced behavioral emergence and cortical activation during SSSA and reduced the depth of anesthesia during deep anesthesia. Therefore, our results demonstrate that SuM glutamatergic neurons play a critical role in regulating states of consciousness under sevoflurane anesthesia.

The SuM is a region in the posterior hypothalamus. Lesions of the posterior hypothalamus can lead to somnolence or catalepsy.^[^ ^25^ ^]^ Electrical stimulation of the SuM can induce cortical arousal.^[^ ^26^ ^]^ Recent studies have reported that SuM glutamatergic neurons are key nodes in controlling wakefulness and that chemogenetic activation of SuM glutamatergic neurons elicits sustained arousal.^[^ ^5^ ^]^ Numerous studies have suggested that some neural substrates are shared between sleep‐wake brain states and general anesthesia.^[^ ^27^ ^]^ Activation of endogenous sleep‐wake pathways contributes to the regulation of recovery from general anesthesia.^[^ ^2^, ^28^ ^]^ However, recent studies found that some brain sites such as the median preoptic nucleus and ventrolateral preoptic nucleus GABAergic or glutamatergic neurons that regulate sleep and wakefulness do not have a substantive influence on anesthetic state transitions.^[^ ^29^ ^]^ Our results suggest that the activation of SuM glutamatergic neurons is sufficient to induce behaviors and cortical activation under sevoflurane anesthesia, which may improve our understanding of the regulation of general anesthesia. However, there is some difference in our studies. Previous studies suggested that activation of SuM glutamatergic neurons promoted wakefulness, whereas inhibition did not alter the sleep‐wake system or the power spectra during waking, although it did reduce theta activity during rapid eye movement sleep.^[^ ^5^ ^]^ In our studies, blocking the activity of SuM glutamatergic neurons significantly facilitated the induction of and delayed behavioral and cortical arousal induced by sevoflurane anesthesia. Thus, the function of SuM glutamatergic neurons may differ in sleep‐wake and anesthesia‐wake states. Moreover, recent studies have suggested that stimulation of SuM neurons reliably drives locomotion and controls locomotion speed.^[^ ^4^ ^]^ It is possible that the combined function of SuM glutamatergic neurons in promoting wakefulness and initiating locomotion may contribute to behavioral arousal during the maintenance of sevoflurane anesthesia. The possible underlying mechanisms need to be further studied.

Glutamatergic neurons in different brain regions may have different effects during sevoflurane anesthesia. Researchers have found that optogenetic stimulation of glutamatergic neurons in the parabrachial nucleus facilitates reanimation from sevoflurane anesthesia.^[^ ^30^ ^]^ Activation of paraventricular thalamic glutamatergic neurons promotes emergence from sevoflurane and propofol anesthesia.^[^ ^6^, ^31^ ^]^ Medial septal glutamatergic neurons have significant arousal‐promoting effects during sevoflueane anesthesia.^[^ ^14^ ^]^ Moreover, activation of glutamatergic neurons in the pedunculopontine tegmental nucleus induces cortical and behavioral arousal under sevoflurane anesthesia.^[^ ^32^ ^]^ Our present results also show that selective stimulation of SuM glutamatergic neurons is sufficient to induce significant behavioral arousal during sevoflurane anesthesia. However, activation of glutamatergic neurons in the lateral habenula accelerates isoflurane anesthesia,^[^ ^33^ ^]^ and blocking glutamatergic output from lateral habenula diminishes the sedative effects of propofol.^[^ ^34^ ^]^ Therefore, exploring the role of glutamatergic neurons and their projections in different nuclei may be important for revealing the mechanism of sevoflurane anesthesia.

Previous studies have suggested a dissociation between cortical activation and behavioral arousal. Only EEG arousal, but not behavioral arousal was induced by microinjection activation of the basal forebrain and optogenetic stimulation of the parabrachial nucleus during anesthesia maintenance.^[^ ^16^, ^30^ ^]^ Conversely, activation of dopaminergic neurons in the ventral tegmental area produces significant behavioral arousal but relatively modest EEG changes during steady‐state isoflurane anesthesia, suggesting that changes in surface EEG may not adequately reflect behavioral wakefulness.^[^ ^10^ ^]^ However, our findings suggest that activation of SuM glutamatergic neurons induces profound arousal in both cortical EEG and behavioral response during steady‐state sevoflurane anesthesia, which was similar to the result obtained from activation of the nucleus accumbens D1 receptor‐expressing neurons as well as activation of medial septal or pedunculopontine tegmental nucleus glutamatergic neurons.^[^ ^14^, ^21^, ^32^ ^]^

Although vesicular glutamate transporter (Vglut2) glutamatergic neurons are mainly distributed within the SuM, this region also contains Vgat, Tac1, and Nos1 neurons.^[^ ^4^, ^5^, ^23^, ^35^ ^]^ Our results showed that both sevoflurane induction and emergence were affected by the activity of SuM glutamatergic neurons, but whether other SuM neuronal subpopulations regulate the effects of sevoflurane anesthesia and how these subpopulations interact with each other remain to be further investigated. Our results confirmed that glutamatergic SuM projection to the MS plays a significant role in arousal from sevoflurane anesthesia. However, it is unclear whether other downstream sites of SuM glutaminergic neurons are associated with general anesthesia in regulating states of consciousness under general anesthesia. The upper brainstem and midbrain components of the arousal system, such as the paraventricular thalamus, locus coeruleus, and basal forebrain, are also likely targets since the SuM has strong connections with these areas.^[^ ^3^ ^]^ Previous studies have suggested that these regions play critical roles under general anesthesia. The activation of paraventricular thalamic glutamatergic neurons promotes the transition from general anesthesia to an arousal state and reduces the recovery time after general anesthesia.^[^ ^6^, ^31^ ^]^ In addition, selective activation of the locus coeruleus noradrenergic system accelerates emergence from general anesthesia and produces cortical activation under deep anesthesia.^[^ ^8^ ^]^ Lesions in locus coeruleus neurons accelerate the induction of and delay the emergence from general anesthesia.^[^ ^36^ ^]^ Moreover, activation of basal forebrain histaminergic neurons induces EEG arousal and facilitates emergence from general anesthesia.^[^ ^16^ ^]^ Stimulation of GABAergic neurons in the basal forebrain promotes behavioral and cortical arousal from isoflurane anesthesia.^[^ ^17^ ^]^ The downstream brain regions involved in the SuM control of arousal from general anesthesia need to be further determined.

EEG delta power is a typical feature of unconsciousness, and delta power is increased during general anesthesia‐induced unconsciousness.^[^ ^37^, ^38^ ^]^ Our results showed that lesioning of SuM glutamatergic neurons could increase delta power during the induction of and emergence from sevoflurane anesthesia, suggesting that the ablation of SuM glutamatergic neurons accelerates LOC during sevoflurane‐induced anesthesia and delays ROC during emergence. In addition, activation of SuM glutamatergic neurons significantly decreased delta power during sevoflurane maintenance, indicating that stimulation of SuM glutamatergic neurons is sufficient to promote the restoration of consciousness during the maintenance of anesthesia. Theta oscillations are associated with exploring an environment or experiencing rapid eye movement (REM) sleep.^[^ ^39^ ^]^ The SuM has long been thought to be a key structure in the brain that generates theta rhythms and silencing of the SuM has been shown to decrease theta power in the hippocampi of anesthetized animals.^[^ ^40^, ^41^ ^]^ However, SuM lesioning failed to abolish hippocampal theta rhythms in freely behaving animals,^[^ ^42^ ^]^ and optogenetic suppression of the SuM did not affect theta rhythms in the CA1 region of the hippocampus, thalamic nucleus reuniens, or medial prefrontal cortex,^[^ ^40^ ^]^ making the effect of the SuM on theta power unclear. Our results showed that lesioning of SuM glutamatergic neurons did not influence theta power during sevoflurane induction, maintenance, or emergence. In addition, alpha power is thought to correspond with levels of consciousness and alpha connectivity is reduced during anesthesia‐induced unconsciousness.^[^ ^43^, ^44^ ^]^ Beta activity is involved in cortex activation and information processing.^[^ ^45^, ^46^ ^]^ Gamma power is involved with awareness during the emergence of general anesthesia.^[^ ^47^ ^]^ Our findings showed that lesioning of SuM glutamatergic neurons significantly decreased alpha, beta, and gamma power during sevoflurane induction and emergence, suggesting that silencing SuM glutamatergic neurons could facilitate LOC during sevoflurane induction and delay ROC during sevoflurane emergence. EEG arousal typically reflects the transition from mixed high‐amplitude slow activity to higher‐frequency low‐amplitude activity. Our present results suggested that the activation of SuM glutamatergic neurons in female mice significantly reduced delta power and increased beta and gamma power during sevoflurane maintenance. However, our results showed that the activation of SuM glutamatergic neurons in male mice decreased alpha activity without affecting beta power and increased only gamma power. One possible explanation is that stimulation of SuM glutamatergic neurons has substantial arousal effects, significantly increasing gamma power, resulting in a decrease in alpha power and no significant increase in beta power.

This study had some limitations. First, this study was conducted with only male mice, and further studies with female animals are needed. Second, we investigated only the times to induction and emergence after lesion of SuM glutamatergic neurons, and further experiments, such as chemogenetic inhibition or neuron excitation, could be performed to strengthen the conclusions.

## 4 Conclusion

In conclusion, these findings enhance our understanding of the mechanisms underlying the effects of sevoflurane anesthesia. Our results suggest that SuM glutamatergic neurons are crucial for behavioral and cortical arousal under sevoflurane anesthesia and offer a potential brain target for optimizing clinical safety and anesthesia management.

## 5 Experimental Section

### Animals

Male Vglut2‐ires‐Cre: Slc17a6^2(cre)Lowl/J^ mice (10‐12 weeks old, weighing ≈22–28 g at the time of the first operation) were group‐housed in a soundproof room (22 ± 1 °C, 50 ± 5% humidity, food and water ad libitum) with an automatic 12‐h light/dark cycle (ZT0/lights on 07:00, illumination intensity ≈100 lux). An online randomization tool (https://www.random.org/lists/) was used to assign animals to different treatment groups. All procedures were approved by the National Animal Care and Use Committee of Sun Yat‐sen University (Guangzhou, China) (protocol number: 2023001057) and were conducted in accordance with the experimental guidelines for animal experimentation of the institutes and Animal Research: Reporting of In Vivo Experiments (ARRIVE) guidelines.

### Viral Vectors and Chemicals

AAV‐EF1a‐DIO‐GCaMP6f and AAV‐Ef1α‐DIO‐mCherry were obtained from BrainVTA (BrainVTA Co., Ltd., Wuhan, China). AAV‐EF1α‐DIO‐taCasp3‐T2A‐TEVp and AAV‐Ef1α‐DIO‐ChR2‐mCherry were obtained from BrainCase (BrainCase Co., Ltd., Shenzhen, China). All viruses were subdivided into aliquots and stored at −80 °C until use. The viral titers ranged from 3 × 10^12^ to 6 × 10^12^ genome copies mL^−1^.

### Surgical Procedures

Mice were anesthetized with sodium pentobarbital (50 mg kg^−1^ i.p.). The eyes were protected with ophthalmic ointment, and the core body temperature was maintained at 37 ± 0.5 °C using a far‐infrared warming pad (RightTemp, Kent Scientific, Torrington, CT, USA) throughout the procedure. Virus injection (20 nL min^−1^) was performed using a glass pipette mounted on a microinjection syringe pump connected to a digital Micro2T controller (Model UMP3T‐2; World Precision Instruments, Sarasota, FL, USA). The injection coordinates in the SuM were as follows: anteroposterior (AP) = −2.92 mm, mediolateral (ML) = ±0.5 mm, and dorsoventral (DV) = −4.9 mm. Following the injection procedure, the glass pipette was left in place for an additional 10 min and was then slowly removed. Three weeks after virus injection, a subset of mice were implanted with surface electrodes on top of the frontoparietal cortex for electroencephalogram (EEG) signal recording, and two electrodes embedded bilaterally in the neck extensor muscles were used to collect electromyogram (EMG) signals. For in vivo fiber photometry recordings and optogenetic manipulations, an optical fiber cannula was implanted above the SuM (AP = −2.92 mm, ML = +0.5 mm, DV = −4.8 mm), and cannulas were secured to the skull with dental adhesive resin cement (C&B‐Superbond, Parkell Inc., Edgewood, NY, USA). For optogenetic manipulations of the SuM‐MS pathway, an optical fiber cannula was implanted above the MS (AP = +0.9 mm, ML = +0.55 mm, DV = −4.2 mm, 8° angle from the midline). The mice were kept warm and monitored until they were fully ambulatory. All mice were allowed to recover for at least two weeks before conditioning.

### Fiber Photometry Recording and Analysis

GCaMP6f fluorescence signals were obtained by a multi‐channel fiber photometry system (Thinker Tech Nanjing Bioscience Inc., China), as described previously.^[^ ^21^, ^32^, ^48^ ^]^ Briefly, following bilateral AAV‐Ef1α‐DIO‐GCaMP6f (2.00E + 12 vg mL^−1^, 200 nl) virus injection into the SuM of Vglut2‐Cre mice, an optical fiber (125 µm O.D., 0.37 numerical aperture; Newton, China) was implanted above the injection site. Four weeks later, the optical fiber was connected to a single‐channel photometry recording system. The fluorescence signals were filtered through a low‐pass filter (40 Hz cutoff; Brownlee 440), digitalized at 500 Hz, and recorded by Spike2 version‐7 software (CED, Cambridge, UK) with simultaneous EEG/EMG recordings. For habituation, the mice were exposed to a cylinder containing 100% oxygen for 120 min on 3 successive days. The Ca^2+^ signals of SuM glutamatergic neurons were recorded during the sevoflurane induction and emergence periods, from wakefulness to loss of consciousness (LOC) or from unconsciousness to recovery of consciousness (ROC), as determined by EEG/EMG recordings. LOC was determined as the moment of a transition from a wake‐EEG state (low‐amplitude, high‐frequency EEG) combined with prominent EMG signals to an anesthesia‐EEG state (high‐amplitude, low‐frequency EEG) combined with low EMG signals. ROC was determined as the moment of a transition to low‐amplitude and high‐frequency EEG with an obvious increase in EMG signals. To analyze the real‐time activities of SuM glutamatergic neurons during sevoflurane induction and emergence, each state transition was first evaluated and then the data was divided according to the EEG/EMG recordings. For sevoflurane induction, the Ca^2+^ signals were analyzed in three phases: baseline (−180 to 0 s, 0 s was the time point at which sevoflurane was initiated), pre‐LOC (the initial period before LOC, from 0 s to LOC) and post‐LOC (the early period after LOC, from LOC to 300 s). For sevoflurane emergence, Ca^2+^ signals were also analysed in three phases: during‐LOC (unconsciousness anesthesia state, −180 to 0 s, 0 s was the time point when sevoflurane was turned off); pre‐ROC (before ROC, from 0 s to ROC); and post‐ROC (the early period after ROC, from ROC to 300 s). To analyze the state transitions during the induction and emergence periods, average signal values were acquired in the corresponding states by integrating ΔF/F_0_. The photometry signal, F, was converted to ΔF/F_0_ = (F−F_0_)/F_0_, where F_0_ is defined as the baseline fluorescence signal during the induction phase (−180 to 0 s, recording period before initiating sevoflurane) or the emergence phase (−180 to 0 s, recording period before turning off sevoflurane).

### Electroencephalogram Recording and Analysis

The EEG/EMG signals were acquired using a tethered data acquisition system at a sampling rate of 500 Hz (Medusa, Biosignal Technologies, Nanjing, China). The EEG signals were analyzed using multitaper methods performed with the Chronux toolbox (http://chronux.org/) in MATLAB 2020a (MathWorks, Cambridge, United Kingdom). EEG power spectra were calculated as previously described.^[^ ^6^ ^]^ Briefly, the original EEG data were analyzed using a 5‐taper fast Fourier transform (FFT) with a window size of 4 s (50% overlap) within a frequency range of 0.5–50 Hz. The power spectral density was calculated for the following frequency bands: delta (0.5–4 Hz), theta (4–8 Hz), alpha (8–15 Hz), beta (15–25 Hz), and gamma (25–50 Hz). The burst suppression ratio was calculated as described previously.^[^ ^49^ ^]^ Briefly, the EEG data were first divided into burst or suppression periods. EEG suppression events were determined based on three conditions: the voltage (amplitude within −20 to 20 µV), duration (minimum duration ≥ 200 ms), and interevent interval (< 50 ms). The period between the suppression events was considered an EEG burst event. EEG burst or suppression events were then assigned values of 0 or 1, respectively, to produce a binary time series. Finally, this binary time series was smoothed with a window function and then computed the burst suppression ratio (BSR) over time.

### Arousal Scoring

Arousal scoring was conducted as described previously.^[^ ^10^, ^21^, ^50^ ^]^ Briefly, spontaneous movements of the limbs, head, and tail were recorded on a detailed score sheet as absent, light movement, or moderate movement (0, 1, or 2, respectively). Furthermore, orienting was scored as 0 if the mouse remained prone and 2 if all four paws of the animal touched the ground. Walking was scored as 0 if the mouse was unable to move after righting, 1 if the mouse crawled but could not raise the abdomen off the floor, or 2 if the mouse walked on four paws with the abdomen off the floor. Overall arousal was calculated as the sum of all categories. The person who performed the scoring was blinded to group allocation.

### Lesion Manipulations

To selectively lesion SuM glutamatergic neurons, AAV‐Ef1a‐DIO‐taCasp3‐TEVP or AAV‐Ef1a‐DIO‐mCherry was bilaterally microinjected into the SuM of Vglut2‐ires‐Cre mice at 20 nL min^−1^. Three weeks later, the mice were acclimatized to a cylindrical chamber containing pure oxygen for 120 min for at least three consecutive days. To assess sevoflurane induction and emergence times, the mice were exposed to 2.4% sevoflurane (1 MAC), and the chambers were rotated 180° every 15 s. The induction time was defined as the duration between anesthesia inhalation and the mice exhibiting loss of the righting reflex at least 30 s after initiating sevoflurane anesthesia. After 30 min of sevoflurane administration, the emergence time was defined as the duration from the cessation of anesthesia inhalation to the return of the righting reflex. The MAC_LORR_ (the concentration at which 50% of the mice lost their righting reflex) and MAC_RORR_ (the concentration at which 50% of the mice recovered their righting reflex) were measured from the dose‐response curve, as described previously. To obtain a dose‐response curve for the LORR, sevoflurane was initially administered at a concentration of 0.7%, and the concentration was increased by 0.2 vol% every 15 min. At the end of each 15‐min interval, the chamber was rotated 180° to explore whether the righting reflex of the animal had disappeared, and the number of animals that lost their righting reflex at each concentration was recorded. The experiments ended when all animals had lost their righting reflex. To determine the dose‐response curve for the RORR, the mice were exposed to 2.1% sevoflurane for 30 min and placed in a supine position. The concentration was decreased by 0.2 vol% every 15 min, and the number of mice that recovered their righting reflex at each concentration was recorded. The sevoflurane concentration was monitored using an anesthetic agent analyzer (G60, PHILIPS, Shenzhen, China). The temperature was maintained at 37 ± 0.5 °C using a heating pad throughout the experiment. The experimenters were blinded to the group allocations.

### Optogenetic Manipulations

To selectively stimulate SuM glutamatergic neurons, AAV‐Ef1α‐DIO‐mCherry or AAV‐Ef1α‐DIO‐ChR2‐mCherry was bilaterally delivered into the SuM of Vglut2‐ires‐Cre mice, and an optical fiber was placed above the SuM. For activating the SuM‐MS projection, AAV‐Ef1α‐DIO‐mCherry or AAV‐Ef1α‐DIO‐ChR2‐mCherry was bilaterally delivered into the SuM of Vglut2‐Cre mice, and an optical fiber was placed above the MS. A power meter (PM100D, Thorlabs, Germany) was used to adjust the blue light intensity at the tip of the optical fiber to 20–30 mW mm^−2^ prior to the behavioral experiments.^[^ ^30^ ^]^ For optical stimulation during maintenance of light sevoflurane anesthesia, the mice were administered 2.5% sevoflurane for 20 min; then, the mice were placed in a supine position. The sevoflurane concentration was then decreased to 1.4%. If the animals showed any signs of regaining their righting reflex, the concentration was increased by 0.1% until the mic lost their righting reflex for at least 20 min at a stable sevoflurane concentration. In this work, the sevoflurane concentration was varied between 1.4% and 1.6%, and optical stimulation (473 nm laser, 5‐ms pulses at 20 Hz for 120 s) was applied to score arousal behavior during steady‐state sevoflurane anesthesia (SSSA). For optogenetic manipulation during deep sevoflurane anesthesia, 2.5% sevoflurane was delivered for 30 min to induce a constant and stable burst suppression pattern, and optogenetic stimulation (473 nm laser, 5 ms pulses at 20 Hz for 120 s) was applied under deep anesthesia.

### Immunocytochemistry

After the experiments, the animals were thoroughly anesthetized and subsequently perfused with 0.01 m phosphate‐buffered saline (PBS), followed by 4% paraformaldehyde (PFA). Post‐infusion, the brains were immersed in 4% PFA overnight for preservation and preserved in 20% and 30% sucrose for dehydration until they sank. The brains were cut into 20 µm segments utilizing a freezing microtome (CM1900, Leica, Wiesbaden, Germany) for immunofluorescence staining of glutamate. Initially, the membrane permeability of the sections was improved using 0.3% Triton X‐100/PBS for 15 min, followed by blocking with 10% goat serum in PBS for an hour at an ambient temperature and overnight incubation at 4 °C with the primary antibody (anti‐glutamate antibody, 1:500, Cat. Case No. G6642, Sigma–Aldrich, USA). Subsequently, the samples were washed with PBS and incubated with the secondary antibody (1:300; goat anti‐rabbit conjugated to Alexa‐488, Invitrogen, USA) for 2 h at room temperature.

### Statistical Analysis

The data were displayed either as the average ± standard deviation (SD) or median ± interquartile range (25th, 75th). All data were evaluated with Shapiro–Wilk normality tests prior to analysis, and parametric or nonparametric tests were selected based on the results. Alterations in neuronal calcium signals were examined using one‐way repeated‐measures ANOVA. Unpaired Student's *t*‐tests were used for lesion experiments. EEG variations in the power spectrum were evaluated using two‐way ANOVA. Using GraphPad Prism 6.0 (GraphPad Software, San Diego, CA, USA), EC50 for the LORR/RORR and the 95% confidence intervals (CIs) based on the dose‐response curves were calculated. A Bayesian Monte Carlo method was used to evaluate the efficacy of photostimulation in restoring the righting reflex during SSSA, as previously described. The posterior probability of the difference in the righting/walking tendency between the ChR2‐on group and the mCherry‐on group was calculated from the beta distribution. Statistical significance was determined based on a posterior probability exceeding 0.95 and Bayesian confidence intervals excluding zero. The BSR was analyzed using two‐way ANOVA with multiple comparisons. In all cases, *P* < 0.05 was considered to indicate a significant difference.

## Conflict of Interest

The authors declare no conflict of interest.

## Author Contributions

J.Y.L., Y.H.W., and Y.H.W. contributed equally to this work. J.Y.L. conceived, designed, and performed the experiments, analyzed/interpreted the data, prepared the figures, and wrote the manuscript. Y.H.W. performed the experiments, analyzed the data, prepared the figures, and wrote the manuscript. Y.H.W. performed the experiments, analyzed the data, and prepared the figures. Y.M.W., R.H., and S.L. performed the experiments and analyzed the data. W.Q.H., L.M. N, and Z.X.W. conceived and designed the experiments as well as wrote and revised the manuscript. All authors read and commented on the manuscript.

## Supporting information



Supporting Information

Supplemental Video 1

Supplemental Video 2

Supplemental Video 3

Supplemental Video 4

Supplemental Video 5

Supplemental Video 6

Supplemental Video 7

Supplemental Video 8

Supplemental Video 9

Supplemental Video 10

## Data Availability

The data that support the findings of this study are available from the corresponding author upon reasonable request.
