# Supplementary material for: Activation of Glutamatergic Neurons in the Supramammillary Nucleus Promotes the Recovery of Consciousness under Sevoflurane Anesthesia
Source: Adv Sci (Weinh). 2025 Apr 1;12(21):2406959. doi: 10.1002/advs.202406959 (PMC12140388; doi:10.1002/advs.202406959)
Supplement: Supplementary file 1 — Supporting Information [file ADVS-12-2406959-s003.docx]

**Supporting information**

**Table S1. Table of best-fit values for sevoflurane dose-response curves** **after lesion of SuM glutamatergic neurons in male mice, Related to Figure 2.** MAC_LORR_ and MAC_RORR_ along with corresponding 95% confidence intervals (CIs) for the control group and the caspase-3 group under sevoflurane anesthesia. MAC_LORR_, minimum alveolar concentration at which 50% of the mice lose their righting reflex; MAC_RORR_, minimum alveolar concentration at which 50% of the mice recover their righting reflex; 95% CI, 95% confidence index.

|  | Chemogenetics lesion of SuM glutamatergic neurons | |
| --- | --- | --- |
|  | Con | Caspase-3 |
| MAC_LORR_, % | 1.55 | 0.76 |
| MAC_LORR_ 95 CI, % | 1.48 to 1.62 | 0.74 to 0.78 |
| Hill’s Slope | 7.89 | 5.50 |
| Hill’s Slope 95 CI | 5.32 to 10.45 | 4.80 to 6.19 |
| MAC_RORR_, % | 1.52 | 0.71 |
| MAC_RORR_ 95 CI, % | 1.31 to 1.75 | 0.67to 0.75 |
| Hill’s Slope | -3.62 | -4.28 |
| Hill’s Slope 95 CI | -4.95 to -2.28 | -5.12 to -3.44 |

**Table S2. Arousal responses under steady-state sevoflurane anesthesia during optogenetic stimulation of the SuM glutamatergic neurons in male mice, related to Figure 4D-F.** Behavioral responses, including spontaneous movements of the limbs, head, and tail, states of the righting reflex and walking, were scored during the 120-s acute optical stimulation of SuM glutamatergic neurons. The total score for each mouse was determined by the sum of all categories.

| ID | Group | Head movement | Leg movement | Tail  movement | Righting | Walking | Total score |
| --- | --- | --- | --- | --- | --- | --- | --- |
| 1 | ChR2-on | 2 | 2 | 2 | 2 | 2 | 10 |
| 2 | ChR2-on | 2 | 2 | 2 | 2 | 2 | 10 |
| 3 | ChR2-on | 2 | 2 | 2 | 2 | 0 | 8 |
| 4 | ChR2-on | 2 | 2 | 2 | 2 | 2 | 10 |
| 5 | ChR2-on | 2 | 2 | 2 | 2 | 2 | 10 |
| 6 | ChR2-on | 2 | 2 | 2 | 2 | 2 | 10 |
| 7 | ChR2-on | 2 | 2 | 2 | 2 | 2 | 10 |
| 8 | ChR2-on | 2 | 2 | 2 | 2 | 2 | 10 |
| 9 | ChR2-on | 2 | 2 | 2 | 2 | 0 | 8 |
| 10 | ChR2-on | 2 | 2 | 2 | 2 | 2 | 10 |
| 1 | mCherry-on | 1 | 1 | 0 | 0 | 0 | 2 |
| 2 | mCherry-on | 0 | 0 | 0 | 0 | 0 | 0 |
| 3 | mCherry-on | 0 | 0 | 0 | 0 | 0 | 0 |
| 4 | mCherry-on | 0 | 0 | 0 | 0 | 0 | 0 |
| 5 | mCherry-on | 1 | 0 | 0 | 0 | 0 | 1 |
| 6 | mCherry-on | 0 | 0 | 0 | 0 | 0 | 0 |
| 7 | mCherry-on | 1 | 0 | 0 | 0 | 0 | 1 |
| 8 | mCherry-on | 0 | 0 | 0 | 0 | 0 | 0 |

**Table S3. Arousal responses under steady-state sevoflurane anesthesia during optogenetic stimulation of the SuM glutamatergic neurons in female mice, related to Figure S1D-F.** Behavioral responses, including spontaneous movements of the limbs, head, and tail, states of the righting reflex and walking, were scored during the 120-s acute optical stimulation of the SuM glutamatergic neurons. The total score for each mouse was determined by the sum of all categories.

| ID | Group | Head movement | Leg movement | Tail  movement | Righting | Walking | Total score |
| --- | --- | --- | --- | --- | --- | --- | --- |
| 1 | ChR2-on | 2 | 2 | 2 | 2 | 2 | 10 |
| 2 | ChR2-on | 2 | 2 | 2 | 2 | 2 | 10 |
| 3 | ChR2-on | 2 | 2 | 2 | 2 | 0 | 8 |
| 4 | ChR2-on | 2 | 2 | 2 | 2 | 2 | 10 |
| 5 | ChR2-on | 2 | 2 | 2 | 2 | 2 | 10 |
| 6 | ChR2-on | 2 | 2 | 2 | 2 | 2 | 10 |
| 7 | ChR2-on | 2 | 2 | 2 | 2 | 2 | 10 |
| 8 | ChR2-on | 2 | 2 | 2 | 2 | 2 | 10 |
| 1 | mCherry-on | 0 | 0 | 0 | 0 | 0 | 0 |
| 2 | mCherry-on | 0 | 0 | 0 | 0 | 0 | 0 |
| 3 | mCherry-on | 0 | 0 | 0 | 0 | 0 | 0 |
| 4 | mCherry-on | 0 | 1 | 0 | 0 | 0 | 1 |
| 5 | mCherry-on | 0 | 0 | 1 | 0 | 0 | 1 |
| 6 | mCherry-on | 0 | 0 | 0 | 0 | 0 | 0 |
| 7 | mCherry-on | 0 | 0 | 0 | 0 | 0 | 0 |

**Table S4. Arousal responses under steady-state sevoflurane anesthesia during optogenetic stimulation of the SuM-MS pathway in male mice, related to Figure 6D-F.** Behavioral responses, including spontaneous movements of the limbs, head, and tail, states of the righting reflex and walking, were scored during the 120-s acute optical stimulation of the SuM-MS pathway. The total score for each mouse was determined by the sum of all categories.

| ID | Group | Head movement | Leg movement | Tail  movement | Righting | Walking | Total score |
| --- | --- | --- | --- | --- | --- | --- | --- |
| 1 | ChR2-on | 2 | 2 | 2 | 0 | 0 | 6 |
| 2 | ChR2-on | 2 | 2 | 2 | 2 | 2 | 10 |
| 3 | ChR2-on | 2 | 2 | 2 | 2 | 0 | 8 |
| 4 | ChR2-on | 2 | 2 | 2 | 2 | 2 | 10 |
| 5 | ChR2-on | 2 | 2 | 2 | 2 | 2 | 10 |
| 6 | ChR2-on | 2 | 2 | 2 | 2 | 2 | 10 |
| 7 | ChR2-on | 2 | 2 | 2 | 0 | 0 | 6 |
| 8 | ChR2-on | 2 | 2 | 2 | 2 | 2 | 10 |
| 1 | mCherry-on | 0 | 0 | 0 | 0 | 0 | 0 |
| 2 | mCherry-on | 0 | 0 | 0 | 0 | 0 | 0 |
| 3 | mCherry-on | 1 | 1 | 0 | 0 | 0 | 2 |
| 4 | mCherry-on | 1 | 0 | 0 | 0 | 0 | 1 |
| 5 | mCherry-on | 1 | 0 | 0 | 0 | 0 | 1 |
| 6 | mCherry-on | 0 | 0 | 0 | 0 | 0 | 0 |

**
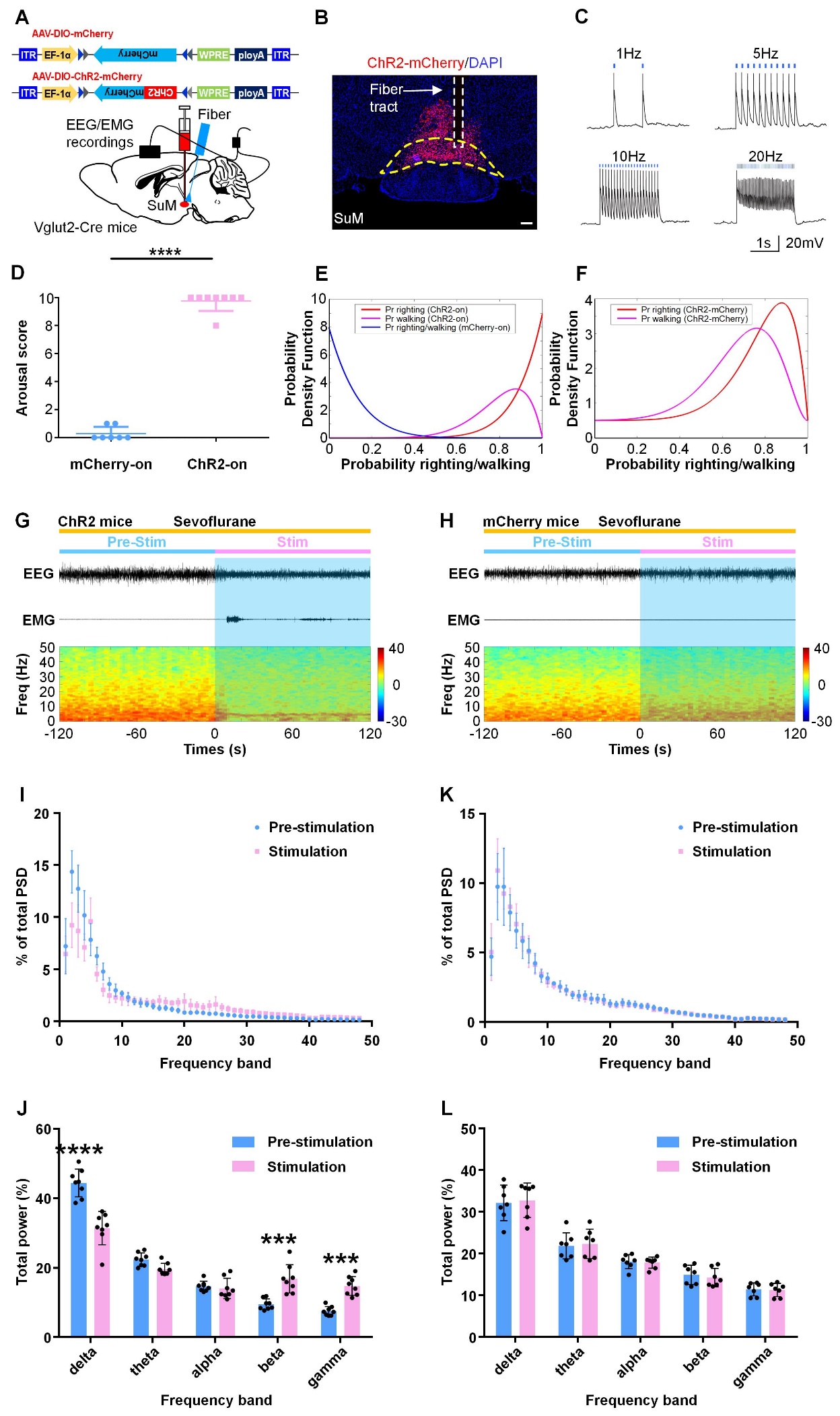
**

**Figure S1.** Optogenetic stimulation of SuM glutamatergic neurons induces arousal behavior and drives cortical activation under steady-state sevoflurane anesthesia in female mice. A) Schematic showing the injection of AAV-DIO-ChR2-mCherry or AAV-DIO-mCherry into the SuM of Vglut2-Cre female mice. B) Tthe expression of ChR2-mCherry (red) in SuM glutamatergic neurons and the position of the optical fiber (white dotted box) in the SuM of female mice. Scale bars, 200 μm. C) Representative traces of neuronal ﬁring in ChR2-expressing glutamatergic neurons in the SuM evoked by 473-nm light stimulation at different frequencies in female mice. D) Arousal scores for the optical activation of SuM glutamatergic neurons (20 Hz, 5 ms pulses, 120 s) in female mice during SSSA. The data are presented as the median ± interquartile range; *********P* < 0.0001, Mann-Whitney U-test, n =7 in the mCherry-on group and n = 8 in the ChR2-on group. E) Posterior distributions for the likelihood of the righting reflex and walking after optical activation of SuM glutamatergic neurons in female mice under SSSA. Posterior distributions were derived from the beta distributions. F) The difference in the posterior probability of the righting reflex and walking between female mCherry-on mice and female ChR2-on mice during SSSA (20 Hz, 5 ms pulses, 120 s). G-H) Typical raw EEG/EMG signals (top) and EEG power spectra (bottom) of ChR2 mice (G) and control mice (H) following acute optical stimulation under SSSA. I) Normalized PSD 120 s before (blue) and 120 s during (pink) stimulation of ChR2 mice during SSSA. J) Relative EEG power before (blue) and during (pink) optical stimulation of SuM glutamatergic neurons. The data are represented as the mean ± SD; ********P* < 0.001, *********P* < 0.0001, two-way repeated-measures ANOVA followed by Sidak’s post hoc test, n = 8 in each group. K) Normalized PSD 120 s before (blue) and 120 s during stimulation of female control mice during SSSA. L) Relative EEG power before (blue) and during (pink) stimulation of control mice during SSSA. The data are represented as the mean ± SD, two-way repeated-measures ANOVA followed by Sidak’s post hoc test, n = 7 in each group.
